# The Association Between Genetic Polymorphisms in Estrogen Receptor Genes and the Risk of Ocular Disease: A Meta-Analysis

**DOI:** 10.4274/tjo.galenos.2020.91298

**Published:** 2020-08-26

**Authors:** Zulvikar Syambani Ulhaq

**Affiliations:** 1 Maulana Malik Ibrahim Islamic State University of Malang, Faculty of Medicine and Health Sciences, Department of Biomedical Science, Batu, Indonesia

**Keywords:** Estrogen receptor, gene, polymorphism, ocular disease

## Abstract

**Objectives:**

To evaluate the association between estrogen receptor *(ER)* genes polymorphisms and the risk of ocular disease.

**Materials and Methods:**

A meta-analysis was performed of all available studies that investigated the association between *ER* gene polymorphisms and the risk of ocular disease.

**Results:**

Studies that were selected based on inclusion criteria reported 5 and 4 single-nucleotide polymorphisms (SNPs) identified in the *ESR1* (ERα) (rs2234093, rs12154178, rs1884054, rs1801132, and rs9340799) and *ESR2* (ERβ) (rs1268656, rs7159462, rs1256031, and rs4986938) genes, respectively. The pooled result showed a significant association between *ESR2* rs1256031 gene polymorphism and ocular disease (odds ratio: 0.55, 95% confidence interval: 0.41-0.74, p<0.0001).

**Conclusion:**

The recessive genotype of *ESR2* rs1256031 gene polymorphism had a protective effect against ocular disease, which supports the hypothesis that the estrogen-signaling pathway through ERβ plays a pivotal role in the pathogenesis of ophthalmic disorders.

## Introduction

Estradiol (E_2_) is a female sex steroid hormone and considered a major form of estrogen.^1^ E_2_ biosynthesis is regulated by rate-limiting enzyme aromatase, which is encoded by the *cyp19a1* gene.^2,3,4,5^ Although the ovary is known as the main site of E_2_ production, local E_2_ synthesis is also observed in several tissues including the brain, adipocytes, bone, liver, and retina.^6,7^ Some evidence suggests an association between gonadal hormones and several diseases that are not related to the reproductive organs, such as diabetes, obesity, and myocardial infarction.^4,8,9^ However, little is known about the role of E_2_ in relation to the eye.

In mammals, expression of *cyp19a1* is detected in the inner nuclear layer, outer plexiform layer, outer nuclear layer, and photoreceptors, while estrogen receptor (ER) is localized in almost all retina layers, cornea, lens, conjunctiva, lacrimal and meibomian glands^7,10^, implying that E_2_ synthesis and its signaling are necessary for the eye. It has been reported that E_2_ exerts a neuroprotective effect in the retina and optic nerve.^11^ Moreover, E_2_ seems to be involved in several eye pathologies such as glaucoma, myopia, age-related maculopathy (ARM), and cataract.^12,13,14,15^ Furthermore, the prevalence of dry eye disease is predominantly found in females, particularly in the menopausal and postmenopausal age group.^16^ Together, these support the notion that estrogen is a contributing factor to the development of ocular disease.

DNA polymorphisms are different DNA sequences that commonly occur among individuals and populations. The most common type of DNA polymorphism is the single nucleotide polymorphism (SNP), which is characterized by a single nucleotide change in the DNA sequence. Interestingly, however, alterations in DNA sequences may be directly or indirectly correlated with the development of disease through the modification of its expression and functional effect.^17^ Estrogen-mediated effects are mainly modulated by ERα and ERβ, which are encoded by the *ESR1* and *ESR2* genes, respectively.^18^ The association between genetic polymorphisms of ER genes and the risk of disease has been reported extensively, particularly in cancer.^19,20^ Two common SNPs have been identified in *ESR1* (rs223469, rs9340799) and *ESR2* (rs1256031, rs4986938).^21,22^ Thus, SNPs in *ESR* genes may directly or indirectly affect normal physiological functions of estrogen and may affect the risk of ocular disease. Therefore, in the present study, a meta-analysis of all eligible studies was performed to provide an accurate estimation of the association between ER polymorphisms and the risk of ocular disease.

## Methods

A literature search was conducted from PubMed, Google Scholar, Scopus, and Web of Science. Keywords such as ER, polymorphisms, and ocular disease were used in combination. The literature search was updated until September 2019. The selection criteria were as follows: (1) evaluating the associations between *ER* gene polymorphisms and the risk of ocular disease and (2) case-control design. The genotypic frequency for the ER polymorphisms was tested by Hardy-Weinberg equilibrium (HWE). Associations between the ER polymorphisms and risk of ocular disease were estimated by calculating pooled odds ratios (OR) and 95% confidence interval (CI). The random-effect model was used to allow heterogeneity. Heterogeneity was evaluated with Q-test and I^2^. Egger’s regression test was used to assess publication bias. P value of <0.05 was indicative of statistical significance.

## Results

From the literature search, 5 studies were selected based on inclusion criteria. The characteristics of the selected studies are shown in Table 1. Mabuchi et al.^12^ recruited 425 glaucoma patients (220 males and 205 females) with the average age of 63.55, while Kosior-Jarecka et al.^21^ enrolled 235 glaucoma patients (72 males and 163 females) with an average age of 75.7. Seitzman et al.^14^ and Imbert et al.^23^ recruited only female participants with an average age of 65 and 62.2, respectively. A study by Škiljić et al.^15^ did not provide information regarding the gender and age of the participants. However, the study evaluated estrogen-related polymorphism in age-related cataracts. All of the studies used age- and gender-matched controls. Thus, this current study mainly consisted of menopausal female participants.

Five and 4 SNPs occurred in the *ESR1* (rs2234093, rs12154178, rs1884054, rs1801132, and rs9340799) and *ESR2* (rs1268656, rs7159462, rs1256031, and rs4986938) genes, respectively. All SNPs complied with the HWE (p>0.05). Finally, only 3 studies were included in our meta-analysis of the association between *ESR1* (rs2234093 and rs9340799) and *ESR2* (rs1256031 and rs4986938) polymorphisms with ocular disease. The pooled results on the association between ER polymorphisms and the risk of ocular disease are shown in Table 2. There was no significant association between *ESR1* gene polymorphisms and risk of ocular disease. However, the recessive model of *ESR2* rs1256031 gene polymorphism showed a 45% decrease in odds ratio (OR: 0.55, 95% CI: 0.41-0.74, p<0.0001, with low heterogeneity I^2^=15%), suggesting a protective effect of recessive genotype in *ESR2* rs1256031 against ocular disease. No publication bias was observed for the association of ER polymorphisms and risk of ocular disease (P_Egger test_>0.05).

## Discussion

In this study, 4 ocular diseases (primary open-angle glaucoma, dry eye, cataract, and ARM) were included in the analysis. Interestingly, such cases are mostly correlated with increased age. Indeed, low estrogen levels are observed in menopausal women and have been associated with increased cytokine production and ocular disease.^23,24^ Moreover, menopausal women treated with hormone replacement therapy show a reduction of intraocular pressure^25,26^, which suggests a protective effect of estrogen. However, there is a conflicting result in regards to *cyp19a1* polymorphism. A woman with *cyp19a1* (rs10046) polymorphism has a higher susceptibility to myopia^24^, while there is no evidence of *cyp19a1* polymorphism being associated with the risk of cataract.^15^ Furthermore, Nishikawa et al.^27^ provided evidence that there is no change in sex steroid hormone levels in patients with vitreoretinal disease. Nonetheless, the role of estrogen on ocular disease needs further investigation.

Because the action of estrogen depends on the interaction with its receptors, understanding ER genetic variations become important to evaluate the association of ER polymorphisms and the risk of ocular disease. This report indicated that the recessive model of *ESR2* rs1256031 gene polymorphism was correlated with a reduction of ocular disease risk, which has been also reported as a protective factor in developing type 2 diabetes mellitus.^28^ Mice lacking ERβ are more susceptible to *in vivo* injury to RPE cells^29^, which supports a protective effect of ERβ. It has been reported that the expression of ERβ is more abundant than ERα in the central nervous system^2,4,30^, including the retina. Moreover, the expression of ERβ is relatively more constant than ERα in the human eye^31^, suggesting a prominent role of ERβ in regulating normal physiological function in the eye.

### Study Limitations

There are limitations to this study. First, the number of studies included in the meta-analysis was relatively small due to the limited availability of published papers. From 5 published papers, only 3 were suitable for further analysis, while the rest evaluated different SNPs. Second, major and minor alleles might have different roles in the risk of ocular disease, resulting in heterogeneity of the studies. Third, although this study was mainly generated from the pooled data of menopausal women, some studies enrolled both male and female participants, which may affect the pooled estimate. Thus, these findings should be interpreted with caution.

## Conclusion

In summary, the current meta-analysis suggests that *ESR2* rs1256031 gene polymorphism is significantly associated with the risk of ocular disease. The recessive genotype of *ESR2* rs1256031 gene polymorphism was associated with a reduced risk of developing ocular disease. It is expected that more studies will become available, which may help the accurate estimation of the relationship of ER with ocular disease to verify this conclusion.

## Figures and Tables

**Table 1:**
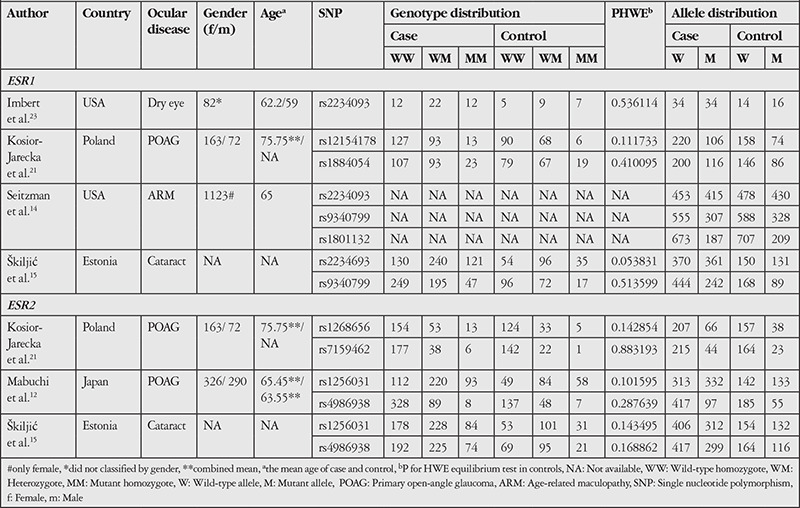
Summary of the studies evaluating the association between ER polymorphisms and the risk of ocular disease

**Table 2:**
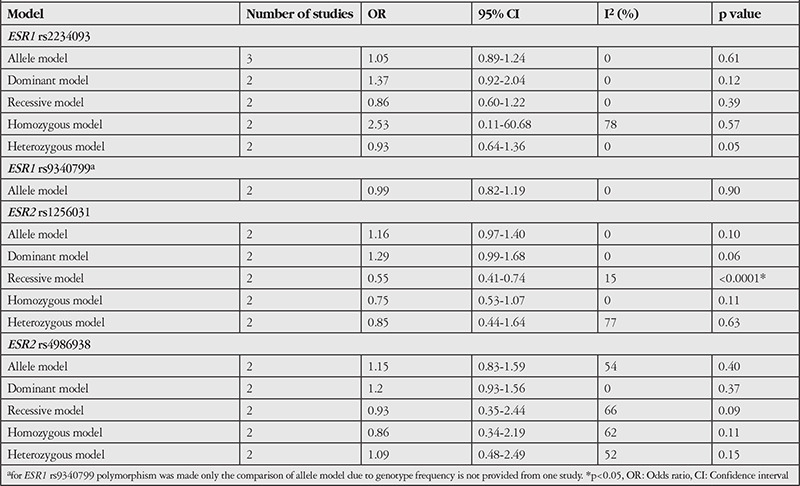
Meta-analysis for the association between ER polymorphisms and the risk of ocular disease
